# A multi-trait epigenome-wide association study identified DNA methylation signature of inflammation among people with HIV

**DOI:** 10.21203/rs.3.rs-4419840/v1

**Published:** 2024-05-31

**Authors:** Junyu Chen, Qin Hui, Boghuma K. Titanji, Kaku So-Armah, Matthew Freiberg, Amy C. Justice, Ke Xu, Xiaofeng Zhu, Marta Gwinn, Vincent C. Marconi, Yan V. Sun

**Affiliations:** Emory University; Emory University; Emory University School of Medicine; Boston University Chobanian and Avedisian School of Medicine; Vanderbilt University School of Medicine and Tennessee Valley Healthcare System; Connecticut Veteran Health System; Connecticut Veteran Health System; Case Western Reserve University; Emory University; Emory University School of Medicine; Emory University

**Keywords:** HIV, EWAS, IL-6, D-dimer, sCD14

## Abstract

Inflammation underlies many conditions causing excess morbidity and mortality among people with HIV (PWH). A handful of single-trait epigenome-wide association studies (EWAS) have suggested that inflammation is associated with DNA methylation (DNAm) among PWH. Multi-trait EWAS may further improve statistical power and reveal pathways in common between different inflammatory markers.

We conducted single-trait EWAS of three inflammatory markers (soluble CD14, D-dimers, and interleukin 6) in the Veteran Aging Cohort Study (n = 920). The study population was all male PWH with an average age of 51 years, and 82.3% self-reported as Black. We then applied two multi-trait EWAS methods—CPASSOC and OmniTest—to combine single-trait EWAS results.

CPASSOC and OmniTest identified 189 and 157 inflammation-associated DNAm sites respectively, of which 112 overlapped. Among the identified sites, 56% were not significant in any single-trait EWAS. Top sites were mapped to inflammation-related genes including *IFITM1, PARP9* and *STAT1*. These genes were significantly enriched in pathways such as “type I interferon signaling” and “immune response to virus”.

We demonstrate that multi-trait EWAS can improve the discovery of inflammation-associated DNAm sites, genes, and pathways. These DNAm sites suggest molecular mechanisms in response to inflammation associated with HIV and might hold the key to addressing persistent inflammation in PWH.

## Introduction

Antiretroviral therapy (ART) has successfully transformed human immunodeficiency virus (HIV) infection from a once-terminal condition into a manageable chronic disease^1^. However, people with HIV (PWH) have an increased risk of a wide range of comorbidities compared to individuals of the same age without HIV infection^2^. This could be due to the persistent inflammation associated with HIV even in PWH who are virologically suppressed on ART. Chronic HIV infection elevates levels of specific inflammatory biomarkers such as interleukin-6 (IL-6), soluble cluster of differentiation 14 (sCD14) and D-dimer, which have been shown to correlate with excess comorbidity risk among PWH with or without ART^3–5^. However, the underlying mechanisms remain largely unknown.

DNA methylation (DNAm) is an epigenetic mechanism that can regulate gene expression without altering the DNA sequence by adding a methyl group, usually at the c5 position of a cytosine that precedes a guanine nucleotide (CpG site). Evidence from current epigenome-wide association studies (EWAS) suggests that DNAm may play an important role in the pathophysiology of inflammation in the general population^6–9^ The genetic and environmental determinants of inflammation could be very different for PWH and could modify the association between inflammation and DNAm^10,11^, thus findings in the general population may not be applicable to PWH. To date, only one EWAS of sCD14 in 1,075 PWH has been conducted and identified 118 CpG sites, 10 of which predicted PWH’s survival time among PWH^12^. One proposed hypothesis behind the association between inflammation and DNAm is that DNAm captures cumulative exposures and physiological responses to inflammation that lead to the variation in disease outcomes in PWH. An alternative hypothesis suggests that DNAm changes in response to HIV infection elevate and maintain the levels of inflammatory markers observed in PWH. The absence of clarity highlights the need for a more extensive and comprehensive study to better understand the DNAm signature of inflammation in PWH.

Many inflammatory markers may share a common physiological pathway and their levels could be intercorrelated. For example, sCD14 can indirectly trigger the release of IL-6^13^. Positive correlations between sCD14 level and D-dimer and between IL-6 and D-dimer have also been reported^14,15^. Nevertheless, current EWAS usually only study one trait at a time, missing the opportunity to systematically incorporate information from multiple available phenotypes. Empirical evidence from genome-wide association studies (GWAS) has suggested that searching for variants associated with multiple traits could improve statistical power^16,17^. A few applications of such multi-trait analysis in EWAS have also demonstrated a similar benefit^18,19^.

We hypothesized that a multi-trait EWAS that jointly analyzes multiple inflammatory markers would enhance statistical power in detecting inflammation-associated DNAm. This approach also aimed to uncover epigenetic pathways shared among various inflammatory markers. In this study, we applied two methods originally developed for GWAS to explore the DNAm signature of three inflammatory markers (IL-6, sCD14 and D-dimers) simultaneously in a cohort of 920 PWH. We identified multiple novel CpG sites that were mapped to inflammation-related genes. Further functional analyses revealed that these genes were enriched in the immune response to viral infection.

## Method

### Study population

We utilized data from the Veterans Aging Cohort Study (VACS), a study that prospectively enrolled veterans with and without HIV at Department of Veterans Affairs medical centers across the United States, matched on age, race/ethnicity, sex, and geographic region^20^. In 2005–2007, 1,525 PWH and 843 people without HIV from the original VACS cohort consented to provide whole blood samples for the VACS Biomarker Cohort (VACS-BC)^21^. The specimens were collected using serum separator and EDTA blood collection tubes, and stored at the central repository at the Massachusetts Veterans Epidemiology Research and Information Center in Boston, Massachusetts^21^.

### Inflammatory markers (independent variables)

Whole blood samples collected from VACS-BC participants were used to measure levels of inflammatory markers including IL-6, sCD14 and D-dimer. IL-6 was measured using the QuantiGlo^®^ IL-6 immunoassay, sCD14 was measured by the Quantikine sCD14 Immunoassay and D-dimer was measured using the Liatest D-DI immunoturbidometric assay. Details of the measurement of these markers can be found in a previous publication^21^. For each marker, raw values that fell outside of the 3 standard deviation boundaries were defined as outliers. Individuals with an outlier value in at least one marker were removed from analyses (n_outlier_=10).

### DNA methylation data (dependent variables)

Blood samples of a subset of participants were selected for genome-wide CpG site profiling using the Illumina Infinium Human Methylation 450K BeadChip^22^. We refer to this subset as VACS-450K (n = 740). Subsequently, due to the availability of a newer-generation DNA methylation array and additional funding, another subset of participants’ blood samples were selected for genome-wide CpG site profiling using the Illumina Methylation EPIC (850K) BeadChip^23^. We refer to this subset as VACS-850K (n = 553). VACS-450K and VACS-850K are mutually exclusive, and the assignment of VACS participants to the two DNA methylation platforms was arbitrary^24^. Associations of DNAm with several outcomes have been consistently replicated in the VACS-450K and VACS-850K groups^12,24^.

Details of DNAm profiling and quality control have been previously described^10,12,24^. Briefly, DNA samples extracted from whole blood were bisulfite-converted, genome-wide amplified, fragmented, purified, hybridized to the arrays, fluorescently stained, and scanned to assess for fluorescence intensities following standard procedures. Intensities were then imported in R for quality control, quantile normalization, and batch correction using the *minfi* R package^25^. DNAm beta values were generated for each CpG site from the processed intensities. CpG sites from both platforms were mapped to Genome Research Consortium human build 37. After all procedures, there were DNAm values for 412,583 CpG sites in VACS-450K, 797,676 CpG sites in the VACS-850K, and 374,642 CpG sites common to both platforms.

### Additional covariates

Demographic and clinical information including age, sex, race/ethnicity, HIV viral load, use of medications, chronic health conditions and opportunistic infections closest to the date of blood sample collection were obtained from the electronic health record. HIV viral load was dichotomized as less than 75 or more than 75 copies/ml. Hepatitis B (HBV) and hepatitis C (HCV) infections were both dichotomized as antibody positive or negative. Smoking and alcohol use were obtained from the VACS survey completed by participants closest to the date of blood collection^26,27^. Six main cell type proportions in whole blood (CD4 + T cells, CD8 + T cells, natural killer T cells, B cells, monocytes and granulocytes) were estimated using cell-type specific CpG sites from a reference panel implemented in the R *minfi* package^28^.

### Final analytical dataset

In the current study, we only included male PWH with complete information for all three inflammatory markers, DNAm, and the additional covariates in VACS-450K (n = 469) and VACS-850K (n = 451), forming the final analytical dataset (n = 920).

### EWAS of single inflammatory markers

Linear mixed effect regression models were utilized to assess the associations between levels of each inflammatory marker and DNAm at individual CpG sites separately in VACS-450K and VACS-850K. Chip IDs were included as a random effect to account for potential batch effects. The regression models were adjusted for age, race, HIV viral load, BMI, HCV, HBV, diabetes, any ART medication, antihypertensive medication usage, cigarette use, alcohol use and calculated cell-type proportions as fixed effects. Summary statistics including beta-coefficients (Beta), standard errors (SE), t-statistics, and p-values were obtained from the linear mixed effect regression models.

In an EWAS of one inflammatory marker within one platform, significance was defined as a Bonferroni corrected p-value less than 0.05. For the 374,642 CpG sites shared between VACS-450K and VACS-850K, meta-analyses were conducted using the inverse variance-based approach in METAL software^29^. Significance in the meta-analyses was defined as having a p-value less than 0.05 after Bonferroni correction for 374,642 testing (nominal p-value < 1.33×10^−7^).

### Multi-trait EWAS

**Figure 1:**
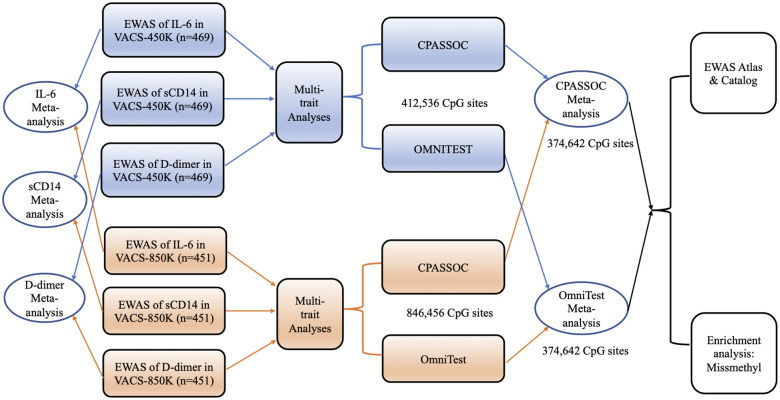
Outline of multi-trait epigenome-wide associations studies of inflammatory markers in the Veteran Aging Cohort Study (VACS).

Overall workflow of the multi-trait EWAS is summarized in Fig. 1. We considered two approaches for multi-trait EWAS. The first one is the test for homogeneous genetic effect across phenotypes in the cross-phenotype association test (CPASSOC), which is essentially a fixed-effect meta-analysis method that can account for the correlations induced by correlated traits measured on the same individuals^30,31^. CPASSOC was originally developed for GWAS data, and it was suggested to use a set of genetic variants not in linkage disequilibrium (LD) to estimate the correlation structure between phenotypes. In our application of CPASSOC to DNAm data, Pearson correlations between inflammatory markers were used to estimate the correlation structure. The CPASSOC package was downloaded from http://hal.case.edu/~xxz10/zhu-web//.

The second approach is an omnibus test (OmniTest) that uses the aggregated Cauchy association test combining p-values from multiple tests, including the minimum of p-values approach (MinP), generalized Berk-Jones test (GBJ), and the generalized higher criticism test (GHC)^32^. MinP accounts for the correlation structures among multiple phenotypes by computing a p-value adjusted for correlated tests (P_ACT_), which is the probability of observing at least one p-value as small as the minimal p-value under the null hypothesis of no association, assuming that the t-statistics for three inflammatory markers follow an asymptotic normality^33^. Both GBJ and GHC were originally designed to test for the association between a set of genetic variants and an outcome while accounting for LD structures^34,35^. In OmniTest, the LD structures are replaced by the correlation structures among inflammatory markers estimated by Pearson correlations.

We considered these two methods because they showed similar performance in a previous study^32^, and we believe they could be complimentary to each other for epigenome-wide discovery. We applied these two approaches to three sets of summary statistics from EWAS of single inflammatory markers, separately in VACS-450K and VACS-850K. A meta-analysis by Fisher’s method was conducted to combine p-values from results in VACS-450K and VACS-850K. Bonferroni corrections were used to adjust for multiple testing in VACS-450K, VACS-850K and meta-analysis separately.

### Associations of identified CpG sites with other phenotypes in the general population

To examine the difference between inflammation-associated DNAm signatures in the general population and PWH, we compared our results to an EWAS of C-reactive protein (CRP) in the general population^36^ in terms of beta-coefficients and p-values. The identified CpG sites from multitrait EWAS in VACS were also searched in the EWAS catalog and EWAS atlas to explore their associations with different disease outcomes in the general population^37,38^.

### Gene set enrichment analysis

We conducted pathway enrichment analysis based on mapped genes from identified CpG sites using the GOmeth function in R package missMethyl^39^. In missMethyl, significant enrichment of Gene Ontology (GO) terms was tested while accounting for the uneven distribution of CpG sites across the genome and CpG sites mapping to more than one gene. We then used REVIGO online software to remove functionally redundant GO terms and visualize the remaining non-redundant GO terms^40^.

## Results

The demographic and clinical characteristics of the study population are summarized in Table 1. Participants in the final analysis dataset were all male and predominantly self-reported Black (82.3%) with an average age of 51.4 (± 7.8). Eighty three percent of the participants were on ART and 53.5% had suppressed HIV viral load (≤ 75 copies/ml). The median levels of IL-6 were 2.1 (Quartile 1–3: 1.5–3.6) pg/mL in VACS-450K and 2.0 (1.4–3.3) pg/mL in VACS-850K. The median levels of sCD14 were similar between the two subsets, 1.7 (1.5–2.1) μg/mL in VACS-450K and 1.7 (1.4–2.1) μg/mL in VACS-850K. The median levels of D-dimer were 0.3 (0.2–0.5) μg/mL in both VACS-450K and VACS-850K.

EWAS of IL-6 identified 43 CpG sites in VACS-450K, 13 CpG sites in VACS-850K, and 97 CpG sites in the meta-analysis (Table 2, Supplementary Fig. 1). EWAS of sCD14 identified 2 CpG sites in VACS-450K, 38 CpG sites in VACS-850K, and 90 CpG sites in the meta-analysis (Table 2, Supplementary Fig. 2). EWAS of D-dimer identified 15 CpG sites in VACS-450K, 16 CpG sites in VACS-850K, and 8 CpG sites in the meta-analysis (Table 2, Supplementary Fig. 3). We showed that the beta-coefficients estimated in VACS-450K and VACS-850K were very comparable for the top CpG sites identified from meta-analyses (Supplementary Fig. 4).

**Figure 2:**
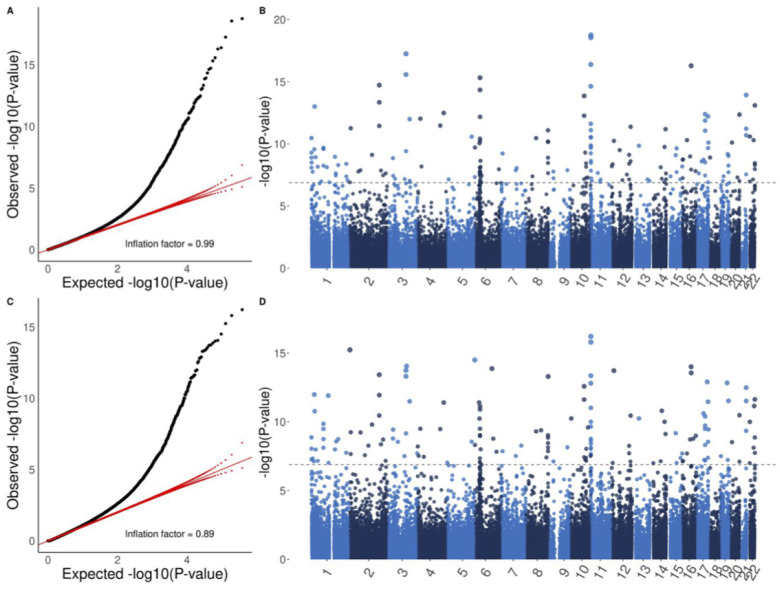
Quantile-quantile (QQ) and Manhattan plots from meta-analyses of multi-trait epigenome-wide associations studies in VACSn450K and VACS-850K. (A) and (B) show the results from the cross-phenotype association test (CPASSOC) while (C) and (D) show the results from the omnibus test (OMNITEST). QQ plots (A and C) compare the observed P-values to the expected P-values. X-axis represents −log10(expected P-values) and Y-axis represents −log10(observed P-values). The red lines indicate the distribution of expected P-values (solid diagonal line) and their 95% confidence interval (dotted lines). The inflation factors estimate the amount of inflation by comparing observed P-values to expected P-values under the hypothesis of no effect. Dashed lines in (B and D) indicate genome-wide significance after adjusting for multiple testing.

Multi-trait EWAS by CPASSOC identified 23, 87 and 189 CpG sites in VACS-450K, VACS-850K and meta-analyses respectively (Table 2, Fig. 2A and 2B, Supplementary Fig. 5). Quantile-quantile (QQ) plots comparing the observed P-values to the expected P-values demonstrated well-controlled inflation (Fig. 2, Supplementary Fig. 5). CPASSOC prioritized CpG sites that had strong associations with all three inflammatory markers, for example, 1-unit increase in IL-6, sCD14 and D-dimer was associated with 0.59%, 4.33% and 2.7% hypomethylation in the most significant CpG site cg03038262 on gene *IFITM1* (CPASSOC P-value = 1.80 × 10^−19^) (Table 3, supplementary Fig. 6). CPASSOC also identified CpG sites that had moderate associations with all three inflammatory markers, such as cg23560388 on gene *TIAM2* and cg12559228 on gene *C19orf76* (Supplementary Table 3, Supplementary Fig. 6). Among the 189 CpG sites identified from meta-analysis of CPASSOC analyses, 74 were not statistically significant in any EWAS of a single inflammatory marker.

OmniTest detected 60, 68, and 157 significant CpG sites in VACS-450K, VACS-850K, and meta-analyses respectively (Table 2, Fig. 2C and 2D, Supplementary Fig. 7). While prioritizing CpG sites associated with all three inflammatory markers, such as cg03038262 (OmniTest P-value = 6.13 × 10^−17^), OmniTest also prioritized CpG sites that were only associated with one or two inflammatory markers. For example, the identification of cg19769520 on gene *PXDN* was mainly its negative association with IL-6 (beta-coefficient = −0.2, P-value = 5.72 × 10^−17^) but not sCD14 (P-value = 0.04) or D-dimer (P-value = 0.02). Among the 157 CpG sites identified from meta-analysis, 42 were not statistically significant in any EWAS of a single inflammatory marker.

**Figure 3:**
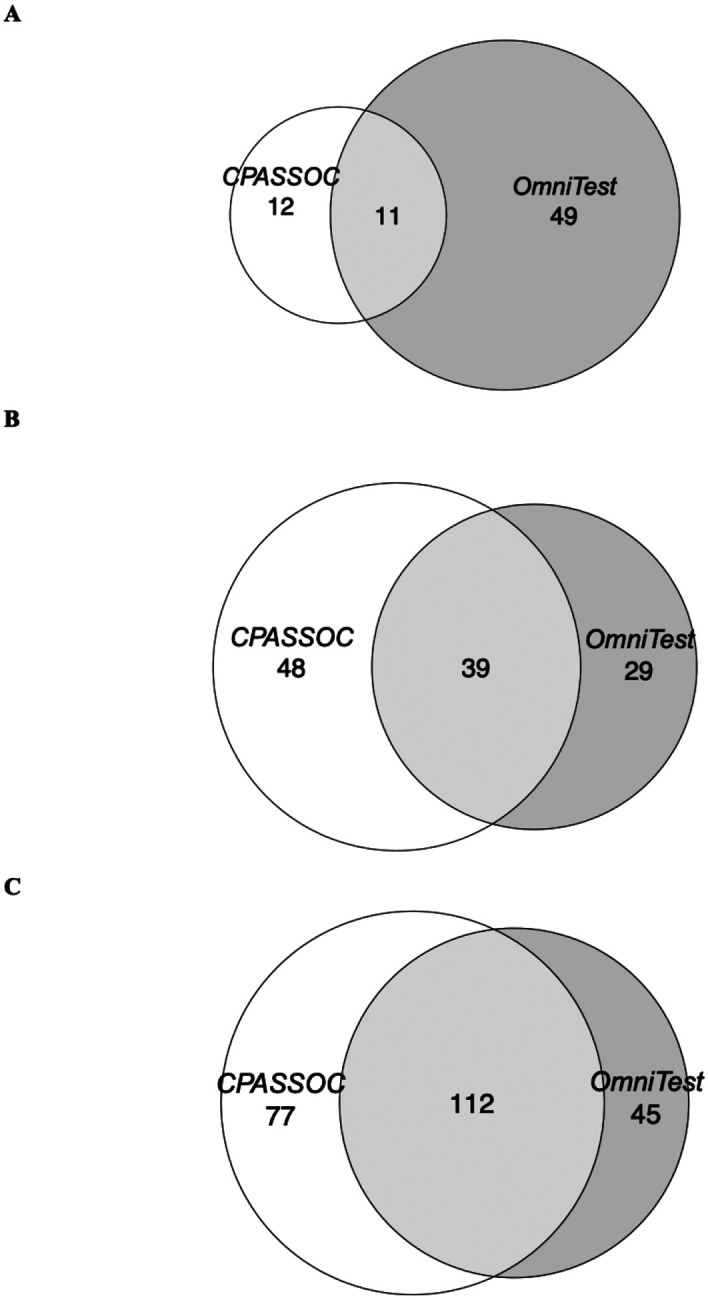
Overlapping CpG sites identified by the cross-phenotype association test (CPASSOC) and the omnibus test (OMNITEST) in (A) VACS-450K, (B) VACS-850K and (C) meta-analyses.

**Figure 4:**
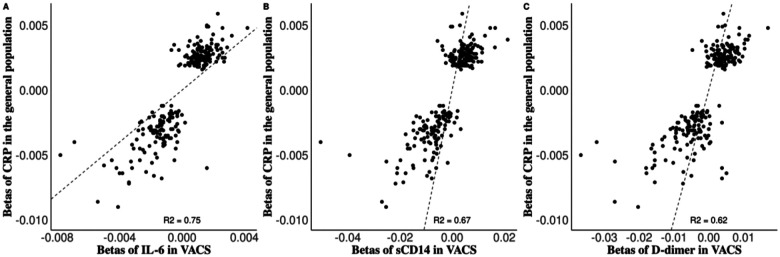
Comparison between the beta-coefficients of the CpG sites associated with (A) IL-6, (B) sCD14, (C) D-Dimer in VACS and the ones associated with C-reactive protein in the general population.

There were 11, 39, and 112 overlapping CpG sites between CpG sites identified from CPASSOC and OmniTest in VACS-450K, VACS-850K and meta-analysis respectively (Fig. 3). The full list of identified CpG sites by CPASSOC and OmniTest are shown in Supplementary Table 1–3. The beta-coefficients for these CpG sites estimated from EWAS of single inflammatory markers showed high correlations (R^2^ > 0.85) (Supplementary Fig. 8), suggesting that changes in methylated cells at these CpG sites are associated with concurrent increase or decrease of all three inflammatory markers. We compared our results to an EWAS of CRP in the general population^36^. One hundred out of 256 (39%) CRP-associated CpG sites in the general population were identified by the multi-trait EWAS in PWH (Supplementary Table 4) and their beta-coefficients were highly correlated with those in single-trait EWAS in PWH (R^2^ > 0.6) (Fig. 4).

**Figure 5:**
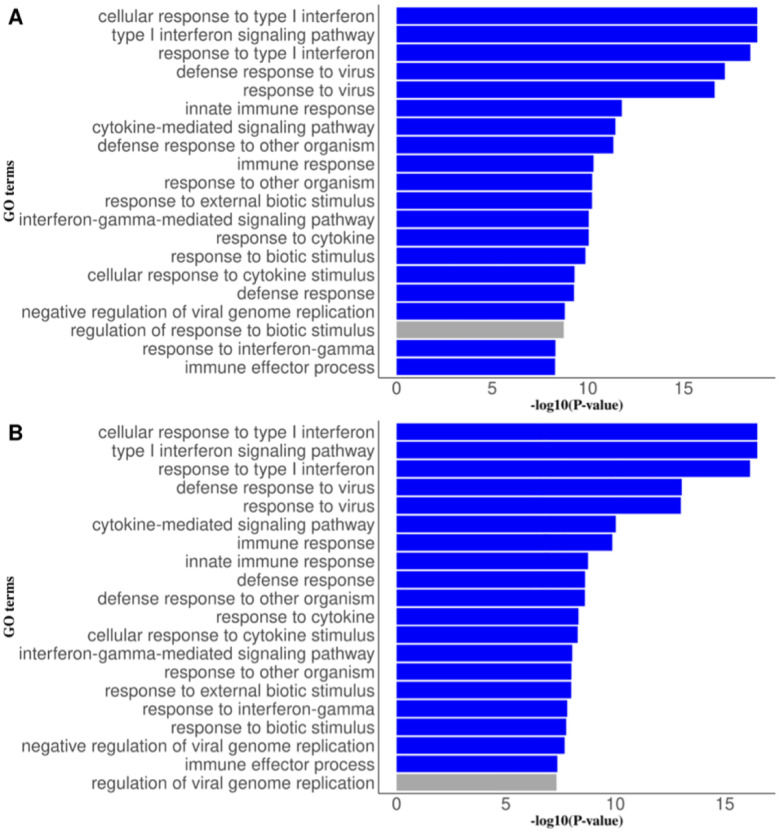
Gene set enrichment analysis by missMethyl using CpG sites identified from meta-analyses of multi-trait epigenome-wide associations studies in VACS-450K and VACS-850K. (A) shows the top 20 GO terms from the cross-phenotype association test (CPASSOC) while (B) shows the top 20 GO terms from the omnibus test (OmniTest). Blue bars indicate consistently significant top 20 GO terms between CPASSOC and OmniTest and grey bars indicate unique top 20 GO terms in each method.

Multiple identified CpG sites were located on the same genes, including genes *ISG15, IFI44L, PLSCR1, TAP1, TAP2, PSMB8, LY6E, FKBP5, IFITM1, IRF7, TMEM49, C19orf76, KLHDC7B, MX1* (Supplementary Table 5). The identified genes were enriched in 72 GO terms, 67 of which are of biological process including the cellular response to type 1 interferon, defense response to virus, innate immune response, cytokine-mediated signaling, response to external biotic stimulus (P-values <5 × 10^−8^) (Fig. 5 and Supplementary Fig. 9). The top 20 GO terms identified by CPASSOC and OmniTest have a large proportion of overlap (> 95%) (Fig. 5).

## Discussion

Our epigenome-wide study jointly analyzed three inflammatory markers and found a total of 295 associations between blood-based DNAm and inflammation in more than 900 male PWH from VACS. Many of these associations were significant in more than one single-trait EWAS, particularly IL-6 and sCD14. By leveraging information from all three inflammatory markers, we identified several CpG sites that were not revealed by any of the single-trait EWAS. The beta-coefficients estimated in the three single-marker EWAS were highly correlated. They were also correlated with beta-coefficients estimated from an EWAS of CRP in the general populations^36^. This suggests concurrent increase or decrease of inflammatory markers might be associated with changes of DNAm at these identified CpG sites.

The leading CpG sites were mapped to genes implicated in immune and antiviral response, such as *STAT1* and *IFITM1*. Many identified genes harbored multiple significant CpG sites, suggesting that CpG methylation in these regions may be regulated concomitantly in response to inflammation in PWH. Many of the identified CpG sites have been reported as associated with at least one autoimmune disease such as rheumatoid arthritis, Crohn’s disease, or multiple sclerosis in the general population^41–44^. The gene-set enrichment analysis based on significant CpG sites also confirmed the role of these genes in inflammatory processes by highlighting the defense response to virus, type-1 interferon signaling, and innate immunity pathways.

Identified genes including *STAT1, IFITM1, IFIT1, IFIT3, MX1, OAS2–3, PARP9, PSMB8, TMEM49, EPSTI1, IFI27, IFI44L, IRF7, ISG20*and *ISG15* are all interferon stimulated genes (ISGs), namely genes whose expression can be robustly up-regulated by interferon signaling^45^. Beta-coefficients for all significant CpG sites located in these ISGs are negative, suggesting that hypomethylation at these CpG sites at ISGs, which usually leads to their higher gene expression, is associated with inflammation. CpG sites on *PARP9, MX1, IFI44L, EPSTI1*, and *TAP1* have been previously reported to be hypomethylated in ART-naive PWH compared to individuals without HIV, which could be related to inflammation in response to HIV infection^46^. In our study, over half of the participants are virally suppressed, and the hypomethylation of the CpG sites at ISGs may be due to oxidative stress triggering a non-specific response, herpesvirus reactivation, gut translocation of bacteria, or persistent viral replication^47^.

The transcription of ISGs is induced by interferon signaling through the Janus kinase (JAK)–signal transducer and activator of transcription (STAT) pathway^48^. The JAK-STAT pathway can both be triggered by and cause inflammation. In the JAK-STAT pathway, STAT1 molecules interact with other STAT proteins and MUC1 to bind to the promoter regions of other ISGs which leads to their increased expression^49,50^. The protein encoded by *IFITM1*, which harbored 10 significant CpG sites in our study, inhibits HIV replication partially through interfering with virus-cell membrane fusion, viral particle infectivity, and viral protein synthesis^51–53^. IFIT proteins can develop a functional complex to restrict viral translation and modulate innate immune signaling^54^. Specifically, IFIT1 molecules bind to an uncapped 5’-ppp on HIV RNA to sequester those RNA while IFIT3 proteins serve to enhance the binding function of IFIT1 molecules^54,55^. Proteins encoded by genes *MX1, OAS2–3, IFI27, IRF7 and ISG20* have also been shown to inhibit HIV viral replication by diverse mechanisms^56–59^. On the other hand, proteins PARP9 and IFI44L may contribute to antiviral responses by mitigating STAT1 molecules in the JAK-STAT pathway^60,61^ while ISG15 functions as a negative regulator of IFN-1 signaling to prevent overt inflammation^62^. A recent study has shown that cells treated with IFITM1-targeted Small interfering RNA (siRNA) cells had attenuated levels of STAT1 and ISG15, suggesting a mediating effect of IFITM1 protein in IFN-γ stimulated protein synthesis^63^. The interferon signaling pathways could be sustaining the persistent inflammation in PWH. Targeting DNAm at the ISGs-mediated pathways may provide new therapeutic avenues for addressing HIV-associated chronic inflammation and its many downstream effects.

Genes outside the ISG group may also contribute the inflammatory process among PWH. For example, cg07839457 located within the promoter region of *NLRCS* was previously shown to be hypomethylated in ART-naïve PWH compared to individuals without HIV, and to become significantly hypermethylated after two years of successful ART, although less so than in individuals without HIV^46^. This hypomethylation has also been observed in adults and children with HIV on ART^22,64^. In our study, we showed that hypomethylation of cg07839457 (*NLRC5*) was linked to IL-6, sCD14 and D-dimer levels. This finding supports the hypothesis that the effects of HIV and ART on DNAm at this specific site, are mediated through inflammation. *NLRC5* encodes a protein that negatively regulates nuclear factor kappa-light-chain-enhancer of activated B cells (NF-κB) and type I interferon signaling pathways^64^, and may serve as a useful target for controlling inflammation in PWH. Further research, ideally with DNAm and inflammatory markers at multiple time points after ART, is needed to elucidate the relationship between DNAm at cg07839457 and persistent inflammation. Other promising genes identified by top CpG sites include *FKBP5*, which promotes NF-κB pathways,^65^ and *KLHDC7B*, which regulates ISGs such as IFITs and STAT1.

Using the EWAS atlas and EWAS catalog, we showed that many of the CpG sites we identified were previously reported as linked to HIV infection, chronic kidney disease, cancer and diverse autoimmune diseases in the general population (Supplementary Table 6). Because many of these disease outcomes have higher prevalence in PWH compared to the general population^66,67^, inflammation could increase HIV comorbidities through DNAm changes at the identified CpG sites. A longitudinal study design that establishes temporality between inflammation, DNAm and HIV comorbidities could help us test these hypotheses and better understand their relationships.

Compared to other methods, CPASSOC and OmniTest combine summary statistics from single-trait EWAS with fewer assumptions about relationships among the three inflammatory markers, which allowed us to treat the inflammatory markers as predictors as in our hypothesis. While we are most confident in the overlapping CpG sites identified by CPASSOC and OmniTest, both methods also provided unique CpG sites to explore due to their different ways of incorporating the correlation structure.

Owing to the unique multi-trait EWAS study design for PWH, we were not able to identify an independent dataset with the same phenotypic and DNAm data in which to replicate our findings. Additionally, our study included only men with HIV and the findings might not be generalizable to women or pediatric population. We used blood-based DNAm in this study and the observed associations might be different in other tissues. Lastly, this study had inflammatory markers measured at only one time point, which may not fully capture the complexity and dynamics of inflammation.

In conclusion, we present evidence that delineates molecular mechanisms associated with HIV-related inflammation through DNAm. We show that hypomethylation at specific CpG sites correlates with increased levels of three key inflammatory markers, making these sites promising targets for mitigating inflammatory responses. Our analyses underscore the importance of ISGs in combating inflammation, which may persist in PWH due to alterations in DNAm. Interestingly, some of these CpG sites have also been linked to diverse inflammation-related phenotypes in the broader population suggesting that a better understanding of the function of these CpG sites could enhance therapeutic strategies for a range of diseases with similar inflammatory pathophysiology.

## Figures and Tables

**Table 1:** Demographic and clinical characteristics of the study population in the Veteran Aging Cohort Study (VACS). Mean ± standard deviation or median (quartile 1 - quartile 3) is reported for continuous variables, and n (%) is reported for categorical variables.

	VACS-450K (n = 469)	VACS-850K (n = 451)	P-value	Total (n = 920)
Age (years)	51.9 (7.9)	51.0 (7.7)	0.033	51.4 (7.8)
Race/Ethnicity			0.004	
White	48.0 (10.2%)	42.0 (9.3%)		90.0 (9.8%)
Black	398.0 (84.9%)	359.0 (79.6%)		757.0 (82.3%)
Hispanic	13.0 (2.8%)	36.0 (8.0%)		49.0 (5.3%)
Other	10.0 (2.1%)	14.0 (3.1%)		24.0 (2.6%)
Any antiretroviral therapy	393.0 (83.8%)	374.0 (82.9%)	0.724	767.0 (83.4%)
Suppressed Viral load (< = 75 copies/ml)	254.0 (54.2%)	238.0 (52.8%)	0.673	492.0 (53.5%)
IL-6 (pg/mL)	2.1 (1.5–3.6)	2.0 (1.4–3.3)	0.378	2.1 (1.4–3.4)
sCD14 (μg/mL)	1.7 (1.5–2.1)	1.7 (1.4–2.1)	0.416	1.7 (1.4–2.1)
D-dimer (μg/mL)	0.3 (0.2–0.5)	0.3 (0.2–0.5)	0.449	0.3 (0.2–0.5)
Body Mass Index (kg/m^2^)	25.8 (4.7)	26.2 (5.0)	0.113	26.0 (4.9)
Diabetes	84.0 (17.9%)	84.0 (18.6%)	0.779	168.0 (18.3%)
Hepatitis B Virus Infection	51.0 (10.9%)	37.0 (8.2%)	< 0.001	88.0 (9.6%)
Hepatitis C Virus Infection	227.0 (48.4%)	158.0 (35.0%)	0.169	385.0 (41.8%)
Current alcohol use	280.0 (59.7%)	276.0 (61.2%)	0.643	556.0 (60.4%)
Current cigarette use	267.0 (56.9%)	246.0 (54.5%)	0.467	513.0 (55.8%)

**Table 2:** Number of CpG sites identified by epigenome-wide association studies (EWAS) of individual-marker and multi-marker EWAS. Successful replication is defined as having adjusted p-value lessthan 0.05 and beta-coefficient in the same direction.

	VAC-450K	VACS-850K	Meta-analysis
EWAS of IL-6	43	13	97
EWAS of sCD14	2	38	90
EWAS of D-dimer	15	16	8
CPASSOC	23	87	189
OMNITEST	60	68	157

**Table 3:** Top 20 CpG sites associated with inflammation markers in meta-analyses of the results from cross-phenotype association analyses (CPASSOC) in VACS-450K and VACS-850K.

CpG site	Chr	Position (bp)	Gene	CPASSOC P-value	Interleukin-6			Soluble CD14			D-dimer		
					Beta	SE	P-value	Beta	SE	P-value	Beta	SE	P-value
cg03038262	11	315262	*IFITM1*	1.80E-19	−0.59	0.09	1.34E-10	−4.33	0.54	7.50E-16	−2.70	0.47	8.68E-09
cg23570810	11	315102	*IFITM1*	2.80E-19	−0.69	0.11	1.52E-10	−5.04	0.64	2.03E-15	−3.22	0.55	6.07E-09
cg22930808	3	122281881	*PARP9*	5.70E-18	−1.11	0.17	1.89E-10	−7.86	1.02	1.56E-14	−4.62	0.89	2.21E-07
cg01971407	11	313624	*IFITM1*	4.09E-17	−0.44	0.07	2.72E-10	−2.96	0.41	5.30E-13	−1.94	0.36	5.73E-08
cg07839457	16	57023022	*NLRC5*	5.23E-17	−0.83	0.13	4.92E-10	−5.86	0.78	4.23E-14	−3.35	0.68	7.77E-07
cg08122652	3	122281939	*PARP9*	2.67E-16	−0.79	0.14	3.41E-08	−6.73	0.84	9.38E-16	−3.43	0.74	3.13E-06
cg00533183	6	32810742	*PSMB8*	4.75E-16	−0.26	0.04	3.93E-10	−1.59	0.25	1.86E-10	−1.11	0.21	2.06E-07
cg00676801	2	191876673	*STAT1*	1.88E-15	−0.24	0.05	7.73E-07	−2.28	0.29	4.99E-15	−1.15	0.25	5.68E-06
cg10552523	11	313478	*IFITM1*	2.33E-15	−0.51	0.09	3.52E-09	−3.76	0.51	1.38E-13	−2.06	0.44	3.29E-06
cg08818207	6	32820355	*TAP1*	4.59E-15	−0.51	0.08	3.65E-10	−3.01	0.48	3.15E-10	−2.05	0.41	6.33E-07
cg22862003	21	42797588	*MX1*	1.17E-14	−0.78	0.14	4.78E-08	−6.46	0.83	5.66E-15	−2.92	0.73	5.96E-05
cg05552874	10	91153143	*IFIT1*	1.37E-14	−0.70	0.12	6.28E-09	−5.47	0.71	1.21E-14	−2.36	0.62	1.43E-04
cg03110996	2	191883483	-	4.49E-14	−0.24	0.05	2.05E-06	−2.06	0.30	1.26E-11	−1.30	0.26	5.95E-07
cg18533225	22	50986813	*KLHDC7B*	7.95E-14	−0.47	0.08	1.98E-08	−3.50	0.48	3.28E-13	−1.71	0.42	5.37E-05
cg08922729	1	21913557	-	9.89E-14	−0.32	0.05	6.41E-11	−1.55	0.29	6.29E-08	−1.19	0.25	1.36E-06
cg05883128	4	169239131	*DDX60*	3.29E-13	−0.63	0.12	7.89E-08	−5.14	0.70	2.11E-13	−2.27	0.61	1.87E-04
cg16936953	17	57915665	*TMEM49*	4.15E-13	−0.54	0.09	8.02E-09	−2.69	0.56	1.35E-06	−2.70	0.47	1.13E-08
cg01190666	20	62204908	*PRIC285*	4.37E-13	−0.29	0.06	1.39E-06	−2.50	0.35	1.19E-12	−1.20	0.31	9.51E-05
cg06188083	10	91093005	*IFIT3*	5.45E-13	−0.58	0.13	4.50E-06	−5.51	0.74	9.28E-14	−2.65	0.65	4.08E-05

Abbreviation: CpG site, cytosine-phosphate-guanine dinucleotide site; Chr, chromosome; bp, base-pair; Beta, Beta-coefficient; SE, Standard error.

**Table 4:** Top 20 CpG sites associated with inflammation markers in meta-analyses of the results from an omnibus test (OMNITEST) in VACS-450K and VACS-850K.

CpG site	Chr	Position (bp)	Gene	OMNITEST P-value	Interleukin-6			Soluble CD14			D-dimer		
					Beta	SE	P-value		SE	P-value	Beta	SE	P-value
cg03038262	11	315262	*IFITM1*	6.13E-17	−0.59	0.09	1.34E-10	−4.33	0.54	7.50E-16	−2.7	0.47	8.68E-09
cg23570810	11	315102	*IFITM1*	1.60E-16	−0.69	0.11	1.52E-10	−5.04	0.64	2.03E-15	−3.22	0.55	6.07E-09
cg19769520	2	1659591	*PXDN*	5.79E-16	−0.20	0.02	5.72E-17	−0.33	0.16	3.54E-02	−0.31	0.13	1.86E-02
cg26312410	5	179743882	*GFPT2*	3.15E-15	−0.09	0.02	2.70E-08	−0.01	0.10	9.28E-01	−0.10	0.09	2.58E-01
cg07290310	3	125741734	*SLC41A3*	8.78E-15	−0.13	0.02	3.51E-08	−0.13	0.14	3.54E-01	−0.23	0.12	5.70E-02
cg07839457	16	57023022	*NLRC5*	9.88E-15	−0.83	0.13	4.92E-10	−5.86	0.78	4.23E-14	−3.35	0.68	7.77E-07
cg07115952	6	109953157	*AKD1*	1.35E-14	−0.13	0.02	8.65E-10	−0.13	0.13	3.22E-01	−0.08	0.11	4.67E-01
cg22930808	3	122281881	*PARP9*	1.85E-14	−1.11	0.17	1.89E-10	−7.86	1.02	1.56E-14	−4.62	0.89	2.21E-07
cg22488164	12	14716910	*PLBD1*	1.92E-14	0.26	0.03	7.36E-15	0.68	0.20	8.43E-04	0.65	0.17	1.75E-04
cg07634706	16	57438524	*CCL17*	2.78E-14	−0.12	0.02	1.56E-08	−0.03	0.12	8.23E-01	−0.11	0.11	2.88E-01
cg00676801	2	191876673	*STAT1*	3.80E-14	−0.24	0.05	7.73E-07	−2.28	0.29	4.99E-15	−1.15	0.25	5.68E-06
cg10552523	11	313478	*IFITM1*	4.43E-14	−0.51	0.09	3.52E-09	−3.76	0.51	1.38E-13	−2.06	0.44	3.29E-06
cg08122652	3	122281939	*PARP9*	4.91E-14	−0.79	0.14	3.41E-08	−6.73	0.84	9.38E-16	−3.43	0.74	3.13E-06
cg10713002	8	144905047	*PUF60*	5.13E-14	−0.12	0.02	2.71E-09	−0.22	0.12	6.70E-02	−0.15	0.10	1.34E-01
cg05014933	17	71537868	*SDK2*	1.26E-13	−0.11	0.02	5.33E-07	−0.01	0.14	9.20E-01	−0.14	0.12	2.27E-01
cg24062310	19	41289994	*RAB4B*	1.48E-13	−0.16	0.02	5.65E-12	−0.16	0.14	2.67E-01	−0.11	0.12	3.83E-01
cg01971407	11	313624	*IFITM1*	1.58E-13	−0.44	0.07	2.72E-10	−2.96	0.41	5.30E-13	−1.94	0.36	5.73E-08
cg05552874	10	91153143	*IFIT1*	2.61E-13	−0.70	0.12	6.28E-09	−5.47	0.71	1.21E-14	−2.36	0.62	1.43E-04
cg22862003	21	42797588	*MX1*	3.25E-13	−0.78	0.14	4.78E-08	−6.46	0.83	5.66E-15	−2.92	0.73	5.96E-05
cg26227957	1	19547285	*KIAA0090*	1.04E-12	0.29	0.04	1.76E-14	0.6	0.23	1.05E-02	0.81	0.20	4.07E-05

Abbreviation: CpG site, cytosine-phosphate-guanine dinucleotide site; Chr, chromosome; bp, base-pair; Beta, Beta-coefficient; SE, Standard error.

## Data Availability

The data that support the findings of this study are available from the corresponding author, YVS, upon reasonable request.
